# WhatsApp-Based Focus Groups Among Mexican-Origin Women in Zika Risk Area: Feasibility, Acceptability, and Data Quality

**DOI:** 10.2196/20970

**Published:** 2021-10-28

**Authors:** Elizabeth Anderson, Mary Koss, Ana Lucía Castro Luque, David Garcia, Elise Lopez, Kacey Ernst

**Affiliations:** 1 Department of Health Promotion Sciences University of Arizona Tucson, AZ United States; 2 International Center for Research on Women Washington, DC United States; 3 El Colegio de Sonora Hermosillo Mexico; 4 Department of Epidemiology and Biostatistics University of Arizona Tucson, AZ United States

**Keywords:** WhatsApp, synchronous text-based focus groups, Zika, Mexican-origin Latinas, social media, mHealth, focus groups, smartphones, mobile phone

## Abstract

**Background:**

Despite unprecedented advances in worldwide access to the internet via smartphones, barriers to engaging hard-to-reach populations remain in many methods of health research. A potential avenue for conducting qualitative research is via participatory web-based media, including the free, popular social platform WhatsApp. However, despite the clear advantages of engaging with participants over a well-established web-based platform, logistical challenges remain.

**Objective:**

This study aims to report evidence on the feasibility and acceptability of WhatsApp as a method to conduct focus groups.

**Methods:**

A pilot focus group was conducted with Spanish-speaking women near the US–Mexico border. The content focus was knowledge and perceived risks for exposure to the Zika virus during pregnancy.

**Results:**

Evidence was obtained regarding WhatsApp as a low-cost, logistically feasible methodology that resulted in rich qualitative data from a population that is often reticent to engage in traditional research. A total of 5 participants participated in a focus group, of whom all 5 consistently contributed to the focus group chat in WhatsApp, which was conducted over 3 consecutive days.

**Conclusions:**

The findings are noteworthy at a time when face-to-face focus groups, the gold standard, are risky or precluded by safe COVID-19 guidelines. Other implications include more applications and evaluations of WhatsApp for delivering one-on-one or group health education interventions on sensitive topics. This paper outlines the key steps and considerations for the replication or adaptation of methods.

## Introduction

### Background

Web-based focus groups are being increasingly used in health research to facilitate or expedite access to hard-to-reach respondents [1]. Compared with traditional, in-person focus groups, web-based platforms, specifically smartphone-based platforms, have lower costs, allow flexible time for participant responses, better protect participant confidentiality, and may increase acceptability in some populations of interest [2]. However, many populations with internet access remain hard to reach for health behavior research, in part because researchers do not use established platforms in which the population is already literate, resulting in limited acceptability [3]. WhatsApp is a chat-based communication app used widely across the globe for one-on-one and large group conversations.

Web- and chat-based focus groups provide rich qualitative data comparable with those collected in traditional in-person focus groups [4], promote more uniform participation rates [5,6], and increase disclosure of personal views, presumably because of greater anonymity than face-to-face methods [7]. Young populations, in particular, increasingly prefer to express themselves using text-based methods sent from their phones [8]. Furthermore, SMS text message–based data collection may even result in more accurate reporting of sexual and other health behaviors than paper-based or voice formats [9]. Social media norms of emotional expression can be qualitatively documented, which mitigates the loss of nonverbal information that would be observed in in-person or video-based focus groups [10-12]. Differences between web-based and in-person focus groups are being eroded as new technologies improve group interaction and the quality of information offered by participants [4]. Despite evidence for this phenomenon in the literature on web-based communication [13], chat-based methods for qualitative data have not been updated accordingly [14,15].

Distinct subgroups of frequent web-based chat users, including young Spanish-speaking Latina women in the United States, may be more likely to participate in research via their preferred web-based medium [13]. For a pilot test of WhatsApp as a focus group platform, we recruited Latina women in a Zika virus risk area (southern Arizona) to assess their knowledge of Zika virus infection in women who are pregnant or may become pregnant. Secondarily, we queried participants’ preferences for receiving health messages on the web. Mexican-origin women in southern Arizona are often difficult to engage with in research because of distrust, population transience related to migratory work patterns, and fears of immigration-related surveillance [16]. High rates of smartphone ownership and WhatsApp use (which is used by 46% of Mexicans every month [17] in lieu of conventional SMS text messaging), especially among younger people, make this group an ideal target population for testing WhatsApp for qualitative research. In addition, wide-reaching cell networks and Wi-Fi coverage in the United States reduce accessibility issues for the purpose of pilot testing.

WhatsApp is a free, globally prevalent mobile app that contributes an estimated 20% of the total time spent on smartphones [18] and allows free instant messaging to individuals or social network groups. It is used less commonly in the United States, where conventional SMS text messaging is more accessible, as are similar app-based group chat platforms, such as Facebook Messenger, GroupMe, and Viber. These apps have variable popularity among age groups, and preferences shift over time. Smartphone users consistently use multiple chat apps with equivalent functionality in idiosyncratic ways, indicating that preferred communication environments can be leveraged for different communication purposes [19]. Interactions on WhatsApp with strangers in special interest groups are commonly accepted [20] and are a key component of social networking [21,22]. Thus, using the platform to engage Mexican-origin participants in focus groups is a potential and highly acceptable method of health data collection. As desired participants likely spend a significant amount of time on WhatsApp regularly (if not daily) already, incorporating a focus group into the platform minimizes participant burden and encourages ongoing participation over several hours or days.

WhatsApp has many other technical and logistical benefits as a qualitative data collection medium. The platform is *end-to-end encrypted*, meaning that a third party cannot decrypt a message even if they are able to access shared data, which is ethically essential to both researchers and participants. The relative anonymity in a chat group with strangers likely increases willingness to discuss sensitive or embarrassing topics; participants can additionally send immediate, direct messages to a group moderator if they want to share a thought but are uncomfortable sending it to the whole group. Users can express themselves with a variety of media ranging from emojis to multiple languages to photos, small image files (eg, gifs), and videos.

### Objective

Despite the potential for using WhatsApp in data collection in a variety of hard-to-reach populations, security and logistical challenges remain to be explored and documented. The purpose of this study is to pilot test the WhatsApp platform as a method of conducting focus groups with Spanish-speaking Latinas and explore its logistical feasibility for broader use. WhatsApp is being increasingly documented in the scientific literature as a useful tool [23-26]. We describe our methods in detail for replication or adaptation.

## Methods

### Context

Environmental conditions in southern Arizona are conducive to a future Zika virus outbreak driven by the rainy season *Aedes aegypti* mosquitos. The Mexican state of Sonora shares a long border with southern Arizona and had the highest number of Zika virus cases in Mexico in 2018 (n=349) [27]. The Zika virus is secondarily transmitted through sex; however, most public health responses have focused exclusively on mosquito-borne transmission, leaving a knowledge gap for women of childbearing age who are at most risk of negative outcomes if infected with the Zika virus. Latinas in the United States have high rates of unintended pregnancy [28], possibly related to low self-efficacy in safe sex negotiation [29] compared with non-Hispanic Whites and may be less equipped to prevent pregnancy to avoid the Zika virus or to use a condom once already pregnant. Therefore, we designed the present focus group guide and corresponding survey to qualitatively explore current Zika virus knowledge, fears about the Zika virus among women who may be pregnant during a future outbreak, and preferences for future public health communications related to Zika virus prevention.

### Preparation for Implementation

Before the study began, in-depth key informant interviews based on a loose script were performed with 6 clinical and administrative members of 2 health care organizations to explore the perceived need for and feasibility of using the WhatsApp platform for collecting information on Zika virus risk in antenatal Latina women. The 2 partnering health care organizations were located in southern Arizona and had prior research relationships with the academic team. Key informants reviewed study materials, including the consent process, proposed focus group questions, and provided feedback on the survey content. Questions were additionally informed by the current literature, as described in the context section above. Interviews were recorded, and the observing researcher (EA) took extensive notes during the interviews. Key informants confirmed that Zika virus awareness programs were ongoing at the target health care organizations, although they were mostly passive and focused on the prevention of mosquito-borne transmission (eg, promoted use of mosquito repellent) with little or no focus on sexual transmission. Key informants also recommended that in-clinic recruitment flyers be provided in English as well as Spanish.

An initial pilot test of the focus group script was conducted in English with graduate student volunteers to remediate any technical issues with WhatsApp. Volunteers additionally tested the enrollment, consent, and survey forms and provided feedback for confusing questions. Qualitative responses of the test focus group with graduate students (n=7) were not recorded except for notes on the process; student volunteers generally provided detailed, high-level commentary on Zika risk as all were health researchers themselves. Bilingual student volunteers offered instrumental feedback on the face validity and quality of interpretation of the focus group questions, enrollment forms, and consent documentation.

### Pilot Study Design

**Figure 1:**
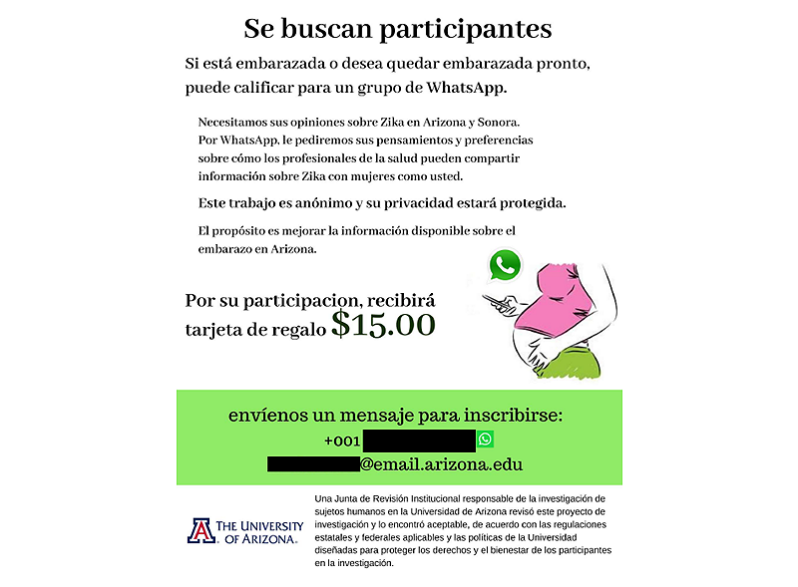
Recruitment flyer for a pilot test of WhatsApp as a focus group platform.

The initial pilot study design involved a multistep recruitment, enrollment, and data collection process. The 2 partnering health care organizations that provided interviews with key informants distributed flyers to Spanish-speaking women of childbearing age seeking care at primary health clinics, prenatal clinics, and Women, Infants, and Children clinics in a region of southern Arizona that is predominantly home to people of Mexican origin. Inclusion criteria were the following: Latina, aged >18 years, and either pregnant or intending to become pregnant in the next year. The flyers included a general study overview, information about compensation for participation, and the WhatsApp contact number of the study coordinator (Figure 1). Clinic staff were briefed on the purpose and methods of the pilot study so that they could answer questions about participation. Approximately 100 flyers were distributed, and the distribution was not limited to women who met the inclusion criteria. Although WhatsApp focus groups may generally be advertisable via social media, this approach would have been unacceptable to the population of interest because of distrust in research as described by the key informants; the partnering clinics and the research projects they endorse are perceived as trustworthy by the population they serve. This study was approved by the University of Arizona institutional review board.

### Enrollment

After the potential participants sent a WhatsApp message to the study coordinator, the coordinator responded with a link to a screening, enrollment, and consent tool on REDCap (Research Electronic Data Capture) hosted at the University of Arizona [30]. REDCap is highly adaptive to all mobile platforms and can be formatted to allow users to toggle between multiple languages on demand; it additionally allows users to save their progress and return to forms at their convenience. Participants were encouraged to consult the study coordinator via WhatsApp for assistance with the enrollment process. Participants entered their email addresses to receive a digital copy of the consent information as well as to receive a digital gift card for their participation later.

### Ensuring Participant Privacy

After successful enrollment, the study coordinator messaged participants to individually counsel about the identifying information that would be collected (ie, phone number and email address). Although the information was included in the consent form, participants were again reminded that other focus group members would be able to see their phone numbers, profile pictures, and public status messages (often consisting of quotes, emojis, personalized messages, or some combination thereof). Participants were reminded to consider the privacy of other participants (ie, they were asked not to take screenshots of group content or contact other group members directly and were asked to report immediately if a phone was lost or stolen while the study was ongoing). Participants were advised to change their profile pictures and not include images of their faces and were offered personalized, step-by-step advice on how to create an anonymous WhatsApp account not linked to their phone numbers (Multimedia Appendix 1 includes instructions for both Android and iOS systems). The study coordinator additionally provided an estimated timeline until the study would begin.

### Focus Group Execution

Focus groups were designed to include between 5 and 7 enrolled participants who selected the same preferred primary language (ie, Spanish or English). A natively bilingual group moderator with graduate-level training in health promotion (DG) was enlisted to conduct the groups. Both the group moderator and the study coordinator communicated with study participants using anonymous WhatsApp accounts to avoid accidental positive identification of study participants by linking to their web-based identities (eg, Facebook’s *people you may know* feature, which links user data such as phone numbers that an individual has contacted to suggest new Facebook friends). The study coordinator created a messaging group within WhatsApp, which included participants, the moderator, and herself.

The group moderator interacted with the participants using a prepared script and a set of primary questions. Given the small sample size, the moderator focused on effective elicitation of the prespecified themes (discussed below) to maximize the utility of the resulting data. The study coordinator followed along and took notes but did not contribute to the group. The group moderator and study coordinator were able to send direct, private messages to one another while the focus group was proceeding, which permitted real-time diagnosis of logistical challenges as well as the ability to discuss key follow-ups and probing questions. Similarly, participants were encouraged to send direct, private messages to the group moderator if they were uncomfortable sharing a thought with the broader group.

### Focus Group Themes

The prepared focus group questions were organized into three themes (general Zika virus knowledge, knowledge about sexual transmission and attitudes toward avoiding sexual transmission, and preferences for internet and WhatsApp use for health messaging), which were delivered (one theme per day) over 3 consecutive weekdays to avoid exhausting the participants. Each day, the start time varied slightly but began in the morning. The purpose of this design was to maximize the time available for each topic, as we anticipated that not all participants would spend time on the app equally. Researchers were able to see which group members had opened any given message, a timestamp for when that message was opened, and when they were last active on WhatsApp. This created data monitoring points for the group moderator to make informed judgments on whether and when to send additional follow-up probes if participants were unresponsive to a given question. At the end of the question set for each day, the group moderator informed participants that data collection for the day was finished but that participants could continue sending messages if they wished and additionally primed participants for the next day’s theme.

### Concluding the Focus Group

**Figure 2:**
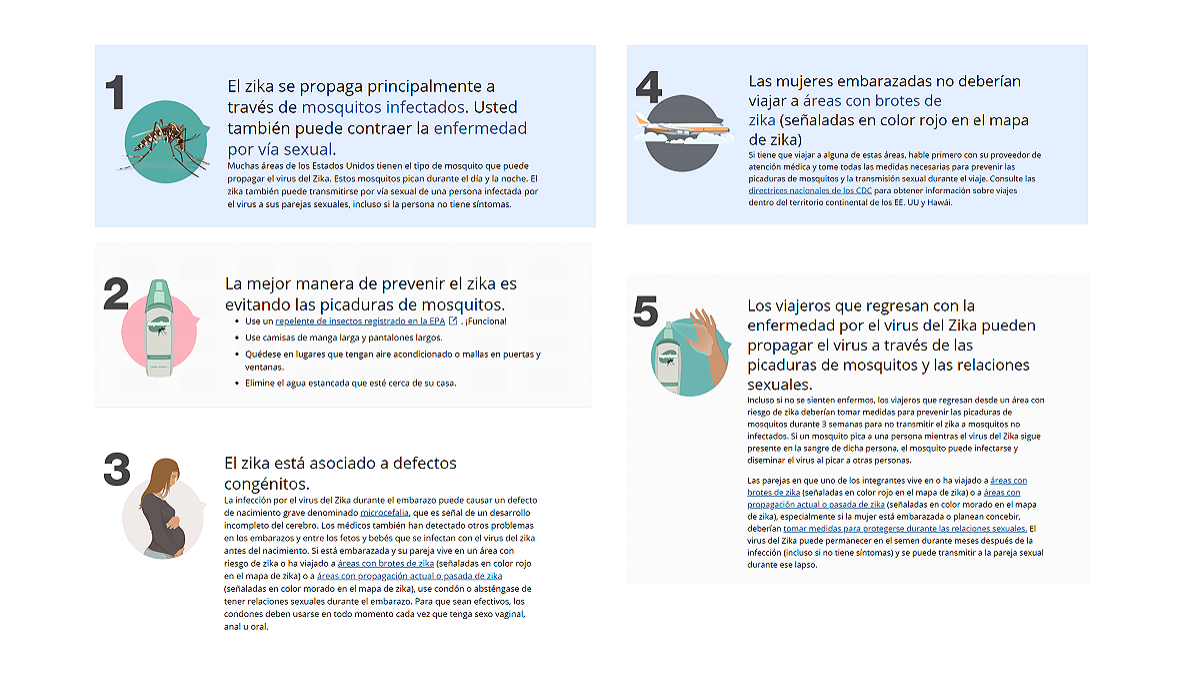
Debrief messages shared as images with participants in a pilot WhatsApp focus group. Source: Centers for Disease Control and Prevention.

After the third day of focus group data collection, the group moderator informed participants that they would receive their gift cards by email. Participants were again encouraged to contact the study coordinator with any questions or concerns and were informed that they would be blocked from the group at the end of the study in order to protect the privacy of fellow participants. The study coordinator sent each participant a private message that consisted of a series of Zika virus infographics in either Spanish or English that were published on the web by the Centers for Disease Control and Prevention (Figure 2). The purpose of this follow-up was to ensure that any misinformation provided by other group members (eg, reported an incorrect Zika virus transmission method, such as by food) would be dispelled.

### Data Processing and Analysis

All data from the focus group were exported from WhatsApp as a text file. To deidentify the data, the study coordinator saved each participant with a code name (eg, FG1 respondent-A) as a phone contact rather than the phone number that was used. Each message was indicated with a timestamp as well as the *name* of the message writer. The file type additionally permitted any emojis to be preserved with no interprogram loss of the image. Any nontext files (eg, voice recordings, screenshots, or shared photos) were additionally included in this export. WhatsApp exports these deidentified files to a variety of web-based locations (eg, Google Drive or Dropbox, or sends them to an email address).

Two researchers (EA and DG) reviewed the transcripts jointly, discussed emergent themes, and agreed upon translations to English. Given the pilot nature of this project as a test of platform feasibility (as well as the small number of participants), the transcript was not systematically coded, and interpretation was limited to a simple thematic analysis with exemplary quotes [31]. We have reported quotes both as originally shared in Spanish as well as their English translations. The reporting of specific results was limited to general observations to protect participant privacy in such a small study.

Corresponding Survey Data Collection

Surveys on demographics, mosquito knowledge, media and technology use, and sexual relationship power were conducted using REDCap to inform the interpretation of focus group data and produce complementary sources of data. Questions were adapted and abridged where appropriate from previous literature to assess the relationship, if any, between focus group responses to similar topic areas and quantifiable knowledge and ability to avoid Zika virus transmission either via mosquitos or from a sexual partner [32], as well as internet use habits [33].

### Alterations in Response to Feedback

After the initial distribution of flyers to the participating clinics, the flyers were revised based on feedback from clinic staff who were promoting the study to patients. Potential enrollees did not initially understand how the study was to be conducted as they had only heard of in-person focus groups. Clinic patients also expressed hesitation about their privacy in the study. Therefore, the flyer was revised to include more information about how the entire study would take place on WhatsApp as well as a more explicit assurance of participant privacy.

Additional changes in the planned methods were made to address key informant feedback. Although we intended to collect survey data at the time of enrollment to streamline the time spent on the REDCap platform, data were collected after the focus groups instead. Our key informants suggested that our intended participants were generally hesitant to share information with perceived authorities because of immigration- and documentation-related fears for themselves or family members. We correspondingly moved the surveys to the end of the process and split the proffered compensation to indicate that responses to the questionnaires were optional (ie, participants received a US $10 gift card for participating in the focus group and an additional US $5 gift card if they selected to complete the REDCap questionnaires, rather than a lump sum of US $15). The questionnaires were linked by REDCap to the participants’ enrollment information by their email addresses.

## Results

### Focus Groups

Of the 7 potential participants who responded to the study coordinator for participation, 5 (71%) were included in a Spanish-language focus group. One enrollee was excluded as she was the only respondent who preferred to participate in English, and one enrollee was excluded as she joined after the first focus group was performed and no additional participants were identified. The recruitment period (September to December) corresponded with the end of the farm work season, meaning that the number of individuals seeking care at the clinic dropped off dramatically shortly after enrollment began; therefore, we terminated enrollment after one focus group was completed at the suggestion of the partnering clinic staff. None of the participants opted to use an anonymous WhatsApp account after receiving the direct message outlining the potential risks of using her personal WhatsApp number.

Respondents consistently participated in the focus group for 3 days (ie, all participants shared at least 2 distinct responses per day [range 2-8], either in response to a question from the moderator or a comment from a fellow participant). The frequency, comprehensiveness, and timing of responses to questions appeared to be highest in the first 2 hours after the group moderator began the daily session. Every respondent provided at least one response per day; the longest delay between the daily initiation and the slowest participant’s response was approximately 2.5 hours. On average, of the 5 respondents, 2 (40%) answered any given question. Respondents who began responding later in the day often began by responding directly to the moderator’s earlier questions and did not feel compelled to skip ahead to the questions that were currently being discussed by other group members. Questions in messages that were opened but did not result in responses were asked again by the group moderator later in the conversation. The overall tone of the conversation was casual, as evidenced by the continued use of slang and internet and text abbreviations commonly used by Mexican-origin Spanish speakers. Approximately 40% (2/5) of participants elaborated on answers using emojis such as these 
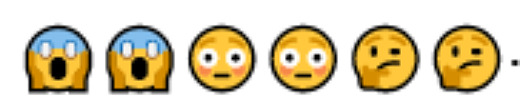
 to express fear (ie, worry about exposure to the Zika virus through mosquito bites) or these 
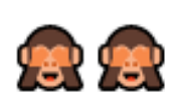
 to communicate the awkwardness explaining why men do not like using condoms.

The median length of responses to focus group prompts was 14 words (range 4-66, IQR 9–22). Responses were concise and demonstrated a clear understanding of the prompt; no responses strayed from the topic of a given prompt, including the longest replies. Evidence that respondents were expressing complex and thoughtful replies was observed where separate ideas or clauses related to the same prompt were shared in back-to-back messages (up to 3 in a row) by the same respondent.

### Corroborating Survey Data

#### Overview

The external survey data shared by individual participants informed the interpretation of focus group responses as the data were linked to respondents by email address. Of the 5 participants, 4 (80%) responded to the optional surveys, indicating that the additional burden of completing the web-based click-through survey was likely not overwhelming for the population. As the sample size was very small, we summarized the most relevant demographic and response trends rather than reporting which survey responses were associated with individual participants: of the 4 respondents, 3 (75%) were currently pregnant, and 3 (75%) had at least 1 previous live birth; all 4 (100%) were married and living with their spouses; 2 (50%) participants indicated that they searched the internet *all the time* for information, including health information such as symptom searches, whereas one-third of participants reported completing such searches several times a week; 3 (75%) participants said they used social media several times a day, though no one said they knew or regularly interacted with strangers on the web; 2 (75%) respondents indicated that they had lower sexual relationship power than their primary male partners, with the other 2 (75%) indicating equal power with their partners. The median age was 29 years; all respondents had finished at least secondary school (high school), and only 1 was currently working.

Below are several examples that illustrate the capacity of WhatsApp focus groups to generate meaningful responses.

#### Theme 1: Zika Virus Knowledge

Focus group participants were in agreement that the Zika virus can influence babies born to infected women (“it’s a virus transmitted by mosquitos and unfortunately mostly affects the baby whose blood gets infected with the virus”) but had mixed knowledge as to what the effects could be (eg, Zika virus “causes paralysis in babies”). Participants knew about mosquito-borne Zika virus transmission, but none had heard of sexual transmission of the virus. There was a general perception that everyone in their southern Arizona community was worried, given that mosquito bite exposure is very common locally:

Respondent C: *A mi en lo personal si me preocupa mucho. Creo q a todos. Por eso trato de siempre usar manga larga y lantalom* [*sic*] *aparte q siempre llevo repelente de mosquitos en mi bolso.*

Respondent C: *pantalon

Respondent C: *For me personally, yes I’m very worried. I think that’s true for everyone. Therefore, I always try to wear long sleeves and lants* [*sic*] *and additionally, I always carry mosquito repellent in my bag.*

Respondent C: *pants

Respondent A: *Yo tampoco miro moskitos dentro d mi hogar y como casi no salgo evitó mucho xk vivo a un lado d un parke y evitamos dejar aguas en botes o estancadas x lo mismo k no se junten mas moscos*

Respondent A: *I also don’t see mosquitos in my house and since I hardly go out b/c I live next to a park, and we avoid leaving water in containers or stagnating so flies don’t gather*

Participants reported using repellents and cleaning up standing water as their primary methods of avoiding exposure to the virus during pregnancy.

#### Theme 2: Sexual and Reproductive Health

When asked about hypothetical medical recommendations to delay becoming pregnant because of Zika virus risk, respondents were hesitant to say that they would be willing to do so for an indefinite period of time:

Moderator: *En algunos países los doctores recomiendan que no queden embarazadas porque no hay tratamiento para el zika, que harían si su doctor les dijera que eviten quedar embarazadas?*

Moderator: *In some countries doctors recommend not becoming pregnant because there is no treatment for Zika, what would you do if your doctor told you to avoid becoming pregnant?*

Respondent B: *Si en verdad quisiera tener un bebé me cuidaría lo más posible de los mosquitos [...] y lo pensaría mucho para ver los pros y cons*

Respondent B: *If I really wanted to have a baby I would take care of mosquitos as much as possible [...] and I would think about it a lot to see the pros and cons*

Moderator: *Qué tal si dice que se esperen 6 meses?*

Moderator: *What if they told you to wait 6 months?*

Respondent B: *Entonces si me espero* 
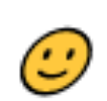

Respondent B: *In that case, yes I’d wait* 
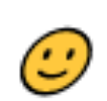

Respondent E: *Si el dr lo recomienda creo que debemos hacer caso ya que ellos son los que saben*

Respondent E: If the dr recommends it, I think we should pay attention as they are the ones who know

Respondents said they had not spoken to their doctors about the Zika virus and that their Zika virus risk conversations with their primary sexual partners were exclusively about mosquito bites, not sexual transmission. When prompted about potentially starting condom use during pregnancy, perceived confidence was consistently high among respondents; 2 suggested that if their partners were reluctant to use a condom, they would show him photos of babies born with microcephaly after maternal Zika virus exposure to change his mind. Participants said they did not know any women whose partners would get mad or suspicious if asked to use a condom during pregnancy and additionally reported that there would be no barriers to getting condoms:

Respondent A: *Creo k nada ps ala pareja ps no le gusta pero a yo pienso k si se trata d cuidarse y d salud eso sale sobrando primero la salud d ambos si esta embarazada ps mas d la d el bebé y si uno esta tratando d salir embarazada ps con musho mas consiencia y cuidarnos para si*

llegara asalir ps todo marche bien primero dios

Respondent A: *I don’t think anything about a couple who wouldn’t like it [to use condoms or other contraceptives], but I think it is about taking care of yourself and your health, that means health first, for both of you if you’re pregnant, but mostly the health of the baby, and if you are trying to get pregnant to be more conscientious and take care of ourselves if we got pregnant, assuming everything goes well first*

#### Theme 3: Technology Use and Preferences

When discussing preferences for receiving Zika virus information from a health professional, most respondents indicated that they would prefer to have a doctor explain prevention methods in person. However, there were mixed responses as to whether some women they knew would prefer to get their information from the news, from friends, or from the internet. Participants commented on a shared concern of receiving false information over the internet and indicated a preference to speak to a health professional, although not necessarily in person:

Respondent A: *Yo si miro o escucho d algo k se está escuchando musho o algo asi en mi siguiente sita selo comento ami doctora*

Respondent A: *If I see or hear something a lot [on WhatsApp or other social media, about Zika] or something like that I just tell my doctor at my next appointment*

## Discussion

### Principal Findings

The pilot WhatsApp focus group consistently involved all participants over multiple days, elicited responses on sensitive topics, included participant interactions that mimicked those seen in traditional focus groups, and privately engaged a population of Spanish-speaking Latinas that is generally hesitant to participate in research. The use of a separate web-based survey to collect data on demographics, knowledge, and attitudes expanded the depth of information without high levels of attrition. Although the focus group provided initial insight into methodological best practices (Textbox 1), positive identification of participants (ie, confirmation that enrollees are who they say they are) remains a challenge. This may be of greater concern when topic areas are sensitive or when the population is more vulnerable to adverse events if their confidentiality is breached. WhatsApp shows initial promise as a focus group platform in a population that already uses the app regularly.

**Best practice takeaways from a pilot test of WhatsApp as a focus group platform.**

- A smaller group (5-7 participants) worked well on the platform though other literature supports larger web-based groups (10-12 participants); more testing is needed.
- Having the study coordinator available to respond quickly to study inquiries and logistical questions about enrollment via WhatsApp was well received and frequently used by participants.
- Conducting the focus group over multiple days seemed to prevent participation fatigue among respondents, who were most responsive to questions early in the day compared with questions asked later in the day.
- Study staff should not link study information to their personal phone numbers or WhatsApp accounts as much as possible, as participants use their phone numbers with other web-based media (eg, Facebook), which could compromise their privacy.
- The burden of "data" use on the target population will likely vary but should be accounted for in the study design.
- It was possible to enroll in the study even if a potential participant did not have an email address; in one case, the study coordinator instructed the participant to enroll, then manually linked her to the survey at the end of the focus group and sent her gift card as a screenshot via WhatsApp, rather than by email.

### Strengths and Weaknesses

In addition to the premise that a WhatsApp-based approach to focus group delivery could engage a reluctant population, we identified several advantages over the course of this pilot study (Textbox 2). For example, the total cost of the pilot group was limited to printing costs, researcher time, and the per-participant cost of gift cards (up to US $15 per person in this study); there were no costs for room rental, equipment rental, snacks, transportation, transcription, software purchases, or other components that are frequently incurred for in-person focus group discussions. However, the weaknesses we identified may merit additional consideration for future implementation on a larger scale or with more vulnerable populations. For example, when a single individual failed to respond to a prompt, it was not possible to distinguish between absence of response because of not having an opinion and not understanding the question. This problem, also present for in-person focus groups, could be mitigated by directly tagging a nonresponding participant in a follow-up prompt to check for understanding.

Although this pilot study spanned 3 days to concur with the 3 parent themes of inquiry, additional testing to identify the optimal length of a WhatsApp focus group is warranted and likely varies among populations and topics. As noted, themes organically overlapped with other days beyond their intended focus, although this would also be expected in a traditional in-person focus group. Overly vocal respondents occur in all types of focus group research; so, in a chat-based setting, it may be that the first voice heard is also the most dominant or that it is simply perceived as such because of the format. As noted, there was a delay of up to several minutes between the time multiple participants opened and read a question and the time the first reply was received, indicating that first repliers were also the most enthusiastic and corresponded with the dominant voices that emerge in traditional in-person focus groups. Chat-based conversation etiquette varies among platforms as well as among populations; therefore, ongoing observation of this phenomenon is warranted for future studies using WhatsApp as described in the present research.

Although we did not identify any safety concerns related to breaches of privacy in our pilot, some potential issues merit consideration if WhatsApp is used for focus groups in other populations. Participants themselves were tasked with determining their level of privacy in the study (eg, decision to use a profile photo of their face, allowing their phone numbers to be seen by other group members, and using the app to discuss sensitive topics on a mobile device that a nonparticipant could potentially access) as well as ensuring the privacy of other participants. The most personal disclosure of sensitive personal information in this study was the inclusion of questions about current intimate partner violence experiences; however, after participants submitted this survey, it was not possible for them (or their intimate partners) to reaccess the answers they had submitted. Although the sensitive topics discussed in this pilot did not likely create a meaningful risk of anticipated or unanticipated breaches of privacy or confidentiality, this may not be the case in smaller subpopulations where participants are more likely to know each other or where inappropriate sharing of information creates a danger to other participants.

**Pros and cons of WhatsApp as a focus group platform identified in a pilot study.**

**Pros**

- It is possible to conduct focus groups without risking the health of the population as may happen in-person gatherings during increasing numbers of COVID-19 cases.
- It maintains continuity of research projects despite stay-at-home orders.
- It allows people from different geographic areas to be recruited to attend. Hard-to-reach populations, including those that are mobile, could be easier to include.
- There are low to no implementation costs.
- Participants do not need to travel, make childcare plans, or adjust their daily schedules to be active respondents to the focus group discussion.
- End-to-end encryption and no face-to-face contact increase the sense of security for those hesitant to engage in research.
- Participants already use WhatsApp and do not need to install a new program or adopt a new behavior in order to be successful, active participants.

**Cons**

- Participants are unfamiliar with the research approach, and additional recruiter training may be needed to ensure accurate study explanations before participants enroll.
- Interactions among the group may limit building upon others' answers, particularly for those that first comment; they may not go back to respond to new comments from participants that interact later in the day.
- Guarantee of privacy is limited to the extent of each participant’s compliance with privacy guidelines, although whether this risk is greater than similar risks posed by in-person research depends on the subject area.
- Highly vulnerable individuals are least likely to own smartphones in many global populations and may be inadvertently excluded from samples.

### Implications for Future Use

Recent research has increasingly identified both intentional and unintentional uses of WhatsApp for health communication [34]. Although most formal evaluations of WhatsApp groups in the literature revolve around its use among health care providers [35], a small randomized controlled trial found that moderated group text discussions in WhatsApp reduced smoking relapse compared with pamphlets alone [36]. However, WhatsApp has also been a medium for the spread of serious misinformation, including around Zika virus transmission (eg, rumors about government conspiracies [37,38]) and vaccine safety [39], indicating a gap in public health education coverage that could be positively leveraged by creating WhatsApp-specific information from demonstrably authoritative sources.

To our knowledge, our pilot study is the first to use WhatsApp for focus group discussions. The potential for expanding access to subpopulations is an important step in data collection. For some global populations, data use can be a prohibitive expense, especially where Wi-Fi is not common; however, WhatsApp uses very little *data* compared with other apps, which is partly why it has been so successful globally. Nevertheless, data use remains a significant consideration for study design, which can often be mitigated by reimbursing participants with phone credit or cash equivalents.

### Future Directions

Beyond focus groups or one-on-one in-depth interviews, WhatsApp could be used to deliver structured educational information to target groups. For example, the population engaged in this pilot study may benefit from structured messages about Zika virus behavior, best practices to avoid mosquito bites, or ways to encourage a partner to consistently use a condom during pregnancy. However, participants in our WhatsApp focus group strongly preferred to receive Zika virus information directly from a health care provider, which may indicate a need to recruit trained community health workers (eg, *promotoras*) who can demonstrate their health authority to participants before intervention delivery.

### Conclusions

This pilot focus group provides a template for using WhatsApp for focus group delivery, as well as initial evidence that WhatsApp is a feasible, low-cost medium for efficient qualitative data enumeration. Innovative methods for distance data collection are in high demand during COVID-19–related restrictions on in-person methods, and low availability of research funding may presage greater future use as well. Additional testing is needed with a wider range of populations and subject matter to broaden the understanding of the risks and benefits to both researchers and participants. Beyond focus groups, WhatsApp has strong potential for use in health promotion research and implementation among global populations with smartphone access, especially where health care professionals are involved.

A full translation of this paper to Spanish is available as Multimedia Appendix 2.

## Appendix

Multimedia Appendix 1 Participant instructions for setting up an anonymous phone number in WhatsApp.

Multimedia Appendix 2 Spanish-language version of the paper.

## Abbreviations

REDCap: Research Electronic Data Capture
