# Supplementary material for: WhatsApp-Based Focus Groups Among Mexican-Origin Women in Zika Risk Area: Feasibility, Acceptability, and Data Quality
Source: JMIR Form Res. 2021 Oct 28;5(10):e20970. doi: 10.2196/20970 (PMC8587330; doi:10.2196/20970)
Supplement: Multimedia Appendix 1 [file formative_v5i10e20970_app1.docx]

Appendix A: Participant instructions for setting up an anonymous phone number in WhatsApp

For both Android and iOS systems:

1. Ensure WhatsApp is already installed.
2. Create and verify an alternative phone number using Google Voice (<https://voice.google.com/>) following the directions provided by Google (available in multiple languages). You must have or create an account with Google in order to set this up. Ensure that text messages sent to your Google Voice number will be forwarded to your smartphone.

For Android:

1. Open the advanced features subfolder under settings. Select the option for Dual Messenger. Turn the dual messenger option “on” for WhatsApp to create a second version of the App. Follow instructions on screen.
2. Open the newly installed secondary version of WhatsApp. When prompted to sign in, do so with the Google Voice phone number. Use the information from the confirmation text to sign in.
3. Toggle between both versions of WhatsApp without signing out of primary account.

For iOS:

1. Sign out of WhatsApp.
2. Sign into WhatsApp using the Google Voice phone number. Use the information from the confirmation text to sign in.
3. It is not possible to toggle between two WhatsApp accounts on an iPhone. Signing out and in between accounts is the only known method to use an anonymous number.

In many global populations it is expected to own and manage multiple SIM cards so providing participants with a brand-new SIM card may be an effective solution to ensuring phone number privacy.
