# Supplementary material for: WhatsApp-Based Focus Groups Among Mexican-Origin Women in Zika Risk Area: Feasibility, Acceptability, and Data Quality
Source: JMIR Form Res. 2021 Oct 28;5(10):e20970. doi: 10.2196/20970 (PMC8587330; doi:10.2196/20970)
Supplement: Multimedia Appendix 2 [file formative_v5i10e20970_app2.docx]

Grupos focales basados ​​en WhatsApp en mujeres de origen mexicano en áreas de riesgo de Zika: un estúdio exploratório sobre la viabilidad, aceptabilidad y calidad de datos

Elizabeth J. Anderson^1^*, Mary Koss^1^, Lucia Castro ^2^, David O. Garcia^1^, Elise Lopez^1^, Kacey Ernst^3^

^1^University of Arizona Department of Health Promotion Sciences, Tucson, AZ

^2^El Colegio de Sonora, Hermosillo, SN, MX

^3^University of Arizona Department of Epidemiology and Biostatistics, Tucson, AZ

*Corresponding Author

1295 N Martin Ave

Tucson, AZ 85712

andersone@email.arizona.edu

**Abstracto**

***Introducción:*** A pesar de avances sin precedentes en el acceso global del Internet a través de teléfonos celulares, siguen existiendo barreras para involucrar a las poblaciones de difícil acceso en muchos métodos de investigación en salud. Una vía posible para realizar investigaciones cualitativas es a través de medios participativos basados ​​en la web, incluyendo la plataforma social, popular y gratuita WhatsApp. Sin embargo, a pesar de las claras ventajas al interactuar con participantes a través de una plataforma web bien establecida, los desafíos logísticos continúan.

***Métodos:*** Este artículo reporta evidencia de la viabilidad y aceptabilidad de WhatsApp como método para llevar a cabo grupos focales con mujeres de habla hispana cerca de la frontera entre los Estados Unidos y México. El contenido se enfoca en el conocimiento y los riesgos percibidos al exponerse al virus del Zika durante el embarazo.

***Resultados:*** Se presenta evidencia de que WhatsApp es una metodología segura, de bajo costo y logísticamente realizable que produce datos cualitativos en una población que es reticente a participar en una investigación tradicional.

***Conclusiones:*** Los resultados son notables en un tiempo cuando el estándar de grupos focales en persona es riesgoso o está excluido de acuerdo las pautas seguras para el COVID-19. Otras implicaciones incluyen una mayor aplicación y evaluación de WhatsApp para ofrecer intervenciones educativas de salud individuales o grupales sobre temas delicados. Este documento describe pasos y consideraciones claves para replicar o adaptar los métodos.

**Introducción**

Se utilizan cada vez más los grupos focales en línea en investigaciones de salud para facilitar o agilizar el acceso a los encuestados de difícil acceso.^1^  En comparación a los grupos focales tradicionales que son en persona, las plataformas en línea –– específicamente las plataformas basadas en teléfonos celulares –– tienen costos más bajos, permiten un tiempo flexible para las respuestas de los participantes, protegen mejor la confidencialidad de los participantes y pueden aumentar la aceptabilidad de algunas poblaciones de interés.^2^ Sin embargo, muchas poblaciones con acceso al Internet siguen siendo difíciles de accesar para investigaciones del comportamiento de la salud, en parte esto es debido a que los investigadores no aprovechan plataformas en las que la población ya utiliza, lo que resulta en una aceptabilidad limitada.^3^

Los grupos focales basados ​​en la web y en el chat resultan en datos cualitativos comparables a los recopilados en los grupos focales tradicionales en persona,^4^ promueven tasas de participación más uniformes,^5,6^ y aumentan la divulgación de puntos de vista personales, presumiblemente debido a un mayor anonimato que los métodos de cara a cara.^7^ Las poblaciones jóvenes, en particular, prefieren expresarse utilizando métodos basados ​​en enviando textos desde sus teléfonos.^8^ Además, la recopilación de datos basada en mensajes de texto puede incluso dar como resultado informes más precisos de comportamientos sexuales y otros comportamientos de salud que los datos obtenidos en papel o basados ​​en formatos de voz.^9^ Las normas de expresión emocional en las redes sociales pueden documentarse cualitativamente, lo que mitiga la pérdida de información que se observaría en grupos focales en persona o en video.^10-12^ Diferencias entre los grupos focales en línea y en persona se están disminuyendo a través de las nuevas tecnologías que mejoran la interacción grupal y la calidad de la información ofrecida por los participantes.^4^ A pesar de la evidencia de este fenómeno en la literatura sobre la comunicación en línea,^13^ métodos basados ​​en chat para datos cualitativos no se han actualizado.^14,15^

Los subgrupos distintos de usuarios frecuentes de chat en línea, incluyendo las jóvenes latinas de habla hispana en los EE. UU., pueden ser más propensos a participar en la investigación a través de su medio preferido basado en la web.^13^ Para una prueba piloto de WhatsApp como plataforma de grupo focal, reclutó a mujeres latinas en un área de riesgo de virus del Zika (sur de Arizona) para evaluar su conocimiento sobre la infección por Zika en mujeres embarazadas o que pueden quedar embarazadas. Les preguntamos por sus preferencias para recibir mensajes de salud en línea. Las mujeres de origen mexicano en el sur de Arizona a menudo son difíciles de involucrar en investigaciones debido a la desconfianza, la fugacidad de la población relacionada con los patrones de trabajo migratorio y los temores a la vigilancia relacionada con la inmigración.^16^ Altas tasas de propiedad de teléfonos celulares y uso de WhatsApp (es usado por el 46% de los mexicanos cada mes^17^ en lugar de mensajes de texto convencionales), especialmente entre las personas jóvenes, hacen de este grupo una población ideal para probar WhatsApp para la investigación cualitativa. Además, la red de amplio alcance de los celulares y la cobertura de Wi-Fi en los EE. UU. reducen los problemas de accesibilidad.

WhatsApp es una aplicación móvil gratuita que prevalece a nivel mundial, contribuye con un estimado del 20% del tiempo total dedicado a los teléfonos celulares^18^ y permite la mensajería instantánea gratuita a individuos o grupos de redes sociales. Se usa con menos frecuencia en los EE. UU., donde el servicio convencional de mensajes [SMS] es más accesible, al igual que las plataformas de chat grupales basadas en aplicaciones similares, como Facebook Messenger, GroupMe y Viber. Estas aplicaciones tienen una popularidad variable entre los grupos de edad y las preferencias cambian con el tiempo. Los usuarios de teléfonos celulares usan constantemente múltiples aplicaciones de chat con una funcionalidad equivalente en formas idiosincrásicas, lo que indica que los entornos de comunicación preferidos se pueden aprovechar para diferentes propósitos de comunicación.^19^ Las interacciones en WhatsApp con extraños en grupos de intereses especiales son aceptadas comúnmente^20^ y son un componente clave de las redes sociales.^21,22^ Por lo tanto, usar la plataforma para involucrar a los participantes de origen mexicano en grupos focales es una forma potencialmente aceptable de recopilación de datos de salud. Debido a que es probable que los participantes deseados ya pasan una cantidad significativa de tiempo en WhatsApp regularmente (si no, diariamente), la incorporación de un grupo de enfoque en la plataforma minimiza la carga de los participantes y fomenta la participación continua durante varias horas o días.

WhatsApp tiene muchos otros beneficios técnicos y logísticos como medio de recopilación de datos cualitativos. La plataforma está "encriptada de extremo a extremo", lo que significa que un tercero no puede descifrar un mensaje incluso si puede acceder a datos compartidos, lo que es éticamente esencial para los investigadores y los participantes. El anonimato relativo de un grupo de chat con extraños probablemente aumenta la disposición a discutir temas delicados o vergonzosos; los participantes también pueden enviar mensajes directos e inmediatos a un moderador de grupo si desean compartir un pensamiento pero se sienten incómodos enviándolo a todo el grupo. Los usuarios pueden expresarse con una variedad de medios desde emojis a múltiples idiomas y fotos, pequeños archivos de imagen (por ejemplo, gifs) y videos.

A pesar del potencial para usar WhatsApp en la recopilación de datos en una variedad de poblaciones difíciles de alcanzar, aún quedan por explorar y documentar los desafíos logísticos y de seguridad. El propósito de este estudio fue probar la plataforma de WhatsApp como método para realizar grupos focales con latinas de habla hispana y explorar su viabilidad logística para un uso más amplio. WhatsApp se documenta cada vez más en la literatura científica como una herramienta útil. Sin embargo, este estudio es, hasta donde sabemos, el primero en adoptar WhatsApp como plataforma de grupo focal. Describimos nuestros métodos en detalle para la replicación o adaptación.

**Metodos**

*Contexto:*

Las condiciones ambientales en el sur de Arizona son propicias para un futuro brote de Zika provocado por la temporada de lluvias de mosquitos *Aedes aegypti*. El estado mexicano de Sonora comparte una frontera larga con el sur de Arizona y tuvo el mayor número de casos de Zika en México en 2018 (n = 346).^23^ El Zika se transmite de forma secundaria a través del sexo, pero la mayoría de las respuestas de salud pública se han centrado exclusivamente en la transmisión a través de los mosquitos, dejando un hueco de conocimiento para las mujeres en edad fértil que corren el mayor riesgo de resultados negativos si están infectadas con Zika. Las latinas en los EE. UU. tienen altas tasas de embarazos no deseados,^24^ posiblemente relacionadas con una autoeficacia baja en la negociación de comportamiento sexual seguro^25^ en comparación con personas que no son hispanas, y pueden estar menos equipadas para prevenir el embarazo y evitar el Zika, o para usar un condón una vez que ya están embarazadas. Por lo tanto, diseñamos una guía de grupos focales y el cuestionario correspondiente para explorar cualitativamente el conocimiento actual sobre el Zika, los temores sobre el Zika entre las mujeres que pueden estar embarazadas durante un brote futuro y las preferencias para futuras comunicaciones de salud pública relacionadas con la prevención del Zika.

*Preparación para la implementación:*

Antes de que comenzara el estudio, se realizaron entrevistas en profundidad por Zoom con informantes clave basadas en un guión con seis miembros clínicos y administrativos de las dos organizaciones de atención médica. Las entrevistas se centraron en la necesidad percibida y la viabilidad de recopilar información sobre el riesgo de Zika en mujeres latinas prenatales. Los informantes clave también revisaron los materiales de estudio, incluido el proceso de consentimiento, las preguntas de los grupos focales y el contenido del cuestionario. Las preguntas también fueron informadas por la literatura actual como se describe en la sección de contexto. Se grabaron las entrevistas y la investigadora observadora (EA) tomó notas extensas durante las entrevistas. Los informantes clave confirmaron que la programación de concientización sobre el Zika estaba en curso con las organizaciones de atención médica, aunque fue principalmente pasiva y se centró en la prevención de la transmisión por mosquitos (por ejemplo, promovió el uso de repelente de mosquitos) con poco o ningún enfoque en la transmisión sexual. Los informantes clave también recomendaron que se proporcionen volantes en la clínica en inglés y en español.

Se realizó una prueba piloto inicial del guión con un grupo focal en inglés con estudiantes voluntarios graduados para remediar cualquier problema técnico con WhatsApp. Adicionalmente, los voluntarios probaron los formularios de inscripción, consentimiento y cuestionario y proporcionaron comentarios en las preguntas que eran confusas. Las respuestas cualitativas del grupo focal de prueba con estudiantes graduados (n = 7) no se registraron, excepto las notas sobre el proceso; los estudiantes voluntarios generalmente proporcionaron comentarios detallados de alto nivel sobre el riesgo de Zika, ya que todos eran investigadores de salud. Los estudiantes voluntarios bilingües ofrecieron retroalimentación instrumental sobre la validez aparente y la calidad de la interpretación de las preguntas del grupo focal, los formularios de inscripción y la documentación de consentimiento.

*Diseño del estudio:*

El diseño inicial del estudio incluyó un proceso de reclutamiento, inscripción y recopilación de datos de varios pasos. Dos organizaciones de atención médica en el sur de Arizona eran socios basados ​​en relaciones de investigación previas con el equipo académico. Distribuyeron volantes a mujeres de habla hispana de edad fértil que buscaban atención en clínicas de salud primarias, clínicas prenatales y clínicas de WIC (mujeres, bebés y niños) en una región del sur de Arizona que es predominantemente hogar de personas de origen mexicano. Los criterios de inclusión fueron: latinas, mayores de 18 años de edad, embarazadas o con intención de quedar embarazadas en el próximo año. Los folletos incluían una descripción general del estudio, información sobre la compensación por la participación y el número de contacto de WhatsApp para la coordinadora del estudio (**Figura 1**). Se informó al personal de la clínica sobre el propósito y los métodos del estudio piloto para que pudieran responder preguntas sobre la participación. Se distribuyeron aproximadamente 100 folletos, aunque la distribución no se limitó a las mujeres que cumplían con los criterios de inclusión. Si bien los grupos focales de WhatsApp generalmente se pueden anunciar a través de las redes sociales, este enfoque habría sido inaceptable para la población de interés debido a la desconfianza en la investigación descrita por los informantes clave; las clínicas asociadas, y los proyectos de investigación que respaldan, son percibidos como confiables por la población. Este estudio fue aprobado por la Junta de Revisión Institucional de la Universidad de Arizona.

Después de la distribución inicial de volantes a las clínicas participantes, los volantes fueron revisados ​​en base a los comentarios del personal de la clínica que promocionaban el estudio a los pacientes. Los inscritos potenciales inicialmente no entendieron cómo se iba a realizar el estudio, ya que solo habían oído hablar de los grupos focales en persona. Los pacientes de la clínica también expresaron dudas sobre su privacidad en el estudio. Por lo tanto, el folleto se revisó para incluir más información sobre cómo se llevaría a cabo todo el estudio en WhatsApp, así como una garantía más explícita de la privacidad de los participantes.

*Inscripción:*

Una vez que los participantes potenciales enviaron un mensaje de WhatsApp a la coordinadora del estudio, la coordinadora respondió con un enlace a una herramienta de detección, inscripción y consentimiento en REDCap (Investigación de Captura Electrónica de Datos) alojada en la Universidad de Arizona.^26^ REDCap es altamente adaptable a todos las plataformas móviles y se pueden formatear para permitir a los usuarios alternar entre varios idiomas; además, permite a los usuarios guardar su progreso y volver a los formularios a su conveniencia. Se alentó a los participantes a consultar a la coordinadora del estudio a través de WhatsApp para obtener ayuda con el proceso de inscripción. Los participantes ingresaron sus direcciones de correo electrónico para recibir una copia digital de la información de consentimiento, así como para luego recibir una tarjeta de regalo digital por su participación.

*Garantizar la privacidad de los participantes:*

Después de una inscripción exitosa, la coordinadora del estudio envió un mensaje a los participantes para asesorar individualmente sobre la información de identificación que se recopilaría (es decir, número de teléfono y dirección de correo electrónico). Aunque se incluyó en el formulario de consentimiento, se les recordó a los participantes que otros miembros del grupo focal podrían ver sus números de teléfono, fotos de perfil y mensajes de estado público (consiste en citas, emojis, mensajes personalizados o alguna combinación de los mismos). Se les recordó a los participantes que consideren la privacidad de otros participantes (es decir, se les pidió que no tomaran capturas de pantalla del contenido del grupo o se contactaran directamente con otros miembros del grupo, se les pidió que informaran inmediatamente si un teléfono se perdió o fue robado mientras el estudio estaba en curso). Se recomendó a los participantes que cambiaran sus imágenes de perfil para no incluir imágenes de sus caras y se les ofreció consejos paso a paso personalizados sobre cómo crear una cuenta anónima de WhatsApp no ​​vinculada a sus números de teléfono (el Apéndice A incluye instrucciones para Android e iOS sistemas). La coordinadora del estudio también proporcionó un cronograma estimado hasta que el estudio comenzará.

*El grupo focal:*

El grupo focal fue diseñado para incluir entre cinco y siete participantes inscritos que seleccionaron el mismo idioma primario preferido (es decir, español o inglés). Una moderadora de grupo nativamente bilingüe con capacitación de nivel de posgrado en promoción de la salud (DG) se alistó para dirigir el grupo. Tanto la moderadora del grupo como la coordinadora del estudio se comunicaron con los participantes del estudio utilizando cuentas anónimas de WhatsApp para evitar la identificación positiva accidental de los participantes del estudio mediante el enlace a sus identidades en línea (por ejemplo, la función "personas que quizás conozcas" de Facebook, que vincula datos de usuario como números de teléfono y la persona se ha contactado para sugerir nuevos amigos de Facebook). La coordinadora del estudio creó un grupo de mensajes dentro de WhatsApp que incluía a los participantes, la moderadora y ella misma.

La moderadora del grupo interactuó con los participantes usando un guión preparado y un conjunto de preguntas principales. Dado el pequeño tamaño del grupo, la moderadora se centró en la obtención efectiva de los temas preespecificados para maximizar la utilidad de los datos resultantes.La coordinadora del estudio siguió y tomó notas, pero no participó directamente. La moderadora del grupo y la coordinadora del estudio pudieron enviarse mensajes directos y privados entre ellas mientras el grupo focal procedía, lo que permitió el diagnóstico en tiempo real de los desafíos logísticos, así como la capacidad de discutir los seguimientos clave y las preguntas de sondeo. Del mismo modo, se alentó a los participantes a enviar mensajes directos y privados a la moderadora del grupo si se sentían incómodos compartiendo un pensamiento con el grupo más amplio.

Las preguntas preparadas del los grupo focal se organizaron en tres temas (conocimiento general sobre el zika; conocimiento sobre la transmisión / actitudes sexuales para evitar la transmisión sexual; y preferencias para el uso de Internet y WhatsApp para mensajes de salud), que se entregaron durante tres días laborables consecutivos para evitar agotar a los participantes. Cada día, la hora de inicio varió ligeramente pero comenzó en la mañana. El propósito de este diseño era maximizar el tiempo disponible para cada tema, ya que anticipamos que no todos los participantes pasarían el tiempo en la aplicación por igual. Las investigadoras pudieron ver qué miembros del grupo habían abierto un mensaje dado, una marca de tiempo de cuándo se abrió ese mensaje y cuándo estuvieron activos por última vez en WhatsApp. Esto permitió que la moderadora del grupo hiciera juicios informados sobre si enviar preguntas de seguimiento adicionales y cuándo enviarlas si los participantes no respondían a una pregunta dada. Al final de la pregunta establecida para cada día, la moderadora del grupo informó a los participantes que la recopilación de datos para el día había terminado pero que los participantes podían continuar enviando mensajes si lo deseaban, y además preparó a los participantes para el tema del día siguiente.

Después del tercer día de recolección de datos del grupo focal, la moderadora del grupo informó a los participantes que recibirían sus tarjetas de regalo por correo electrónico. Se alentó nuevamente a los participantes a contactar a la coordinadora del estudio con cualquier pregunta o inquietud y se les informó que serían bloqueados del grupo al final del estudio para proteger la privacidad de los demás participantes. La coordinadora del estudio envió a cada participante un mensaje privado que consistía en una serie de infografías sobre el Zika en español o inglés que fueron publicadas en línea por los Centros para el Control y la Prevención de Enfermedades (CDC) (**Figura 2**). El propósito de este seguimiento fue asegurar que cualquier información errónea proporcionada por otros miembros del grupo (por ejemplo, que informara un método de transmisión de Zika incorrecto, como por ejemplo por comida) sería disipada.

Todos los datos del grupo focal se exportaron desde WhatsApp como un archivo de texto. Para desidentificar los datos, el coordinador del estudio guardó a cada participante con un nombre en clave (por ejemplo, FG1_respondent-A) como contacto telefónico en lugar del número de teléfono que se utilizó. Cada mensaje se indicó con una marca de tiempo, así como el "nombre" del escritor del mensaje. El tipo de archivo adicionalmente permitió que cualquier emojis se conservará sin pérdida de la imagen entre programas. Cualquier archivo que no sea de texto (por ejemplo, grabaciones de voz, capturas de pantalla o fotos compartidas) se incluyó adicionalmente en esta exportación. WhatsApp exporta estos archivos no identificados a una variedad de ubicaciones en línea (por ejemplo, Google Drive o Dropbox, o los envía a una dirección de correo electrónico).

Dos investigadoras (EA y DG) revisaron la transcripción conjuntamente, discutieron temas emergentes y acordaron traducciones al inglés. Dada la naturaleza piloto de este proyecto como prueba de la viabilidad de la plataforma (así como el pequeño número de participantes), la transcripción no se codificó sistemáticamente y la interpretación se limitó a un simple análisis temático con citas ejemplares.^27^ Reportamos las citas como se compartieron originalmente en español y sus traducciones al inglés. Para proteger la privacidad de los participantes en un estudio tan pequeño, el informe de resultados específicos se limitó a observaciones generales.

*Recopilación de datos de cuestionarios correspondientes:*

Para informar la interpretación de los datos del grupo focal y producir fuentes complementarias de datos, se realizaron cuestionarios sobre el perfil demográfio, conocimiento de mosquitos, uso de medios y tecnología, y poder de relación sexual utilizando REDCap. Las preguntas fueron adaptadas y resumidas, según corresponda, de la literatura anterior para evaluar la relación, si la hubiera, entre las respuestas del gruop focal a áreas temáticas similares y el conocimiento cuantificable y la capacidad de evitar la transmisión del Zika a través de mosquitos o de una pareja sexual,^28^ así como los hábitos del uso del Internet.^29^ Se hicieron algunos cambios en los métodos planificados para abordar los comentarios de los informantes clave. Aunque teníamos la intención de recopilar datos de la cuestionario en el momento de la inscripción para optimizar el tiempo dedicado a la plataforma REDCap, los datos se recopilaron después del grupo focal. Los informantes clave sugirieron que nuestra muestra selecionada generalmente dudaba en compartir información con las autoridades percibidas relacionadas con la inmigración y los temores de documentación para ellos mismos o los miembros de su familia. De manera correspondiente, trasladamos ls encuestas al final del proceso y dividimos la compensación ofrecida para indicar que la respuesta a los cuestionarios era opcional (es decir, los participantes recibieron una tarjeta de regalo de $10 por participar en el grupo focal y una tarjeta de regalo adicional de $5 si seleccionaban completar los cuestionarios de REDCap, en lugar de una suma global de $15). REDCap vinculó los cuestionarios con la información de inscripción de los participantes mediante sus direcciones de correo electrónico.

**Resultados**

*El grupo focal:*

De siete participantes potenciales que respondieron a la coordinadora del estudio para participar, cinco fueron incluidos en un grupo focal en español. Se excluyó a una persona inscrita porque ella era la única encuestada que prefería participar en inglés; se excluyó a una persona inscrita porque se unió después de que se realizó el primer grupo focal y no se identificaron participantes adicionales. El período de reclutamiento (septiembre a diciembre) correspondió con el final de la temporada de trabajo agrícola, lo que significa que la cantidad de personas que buscaban atención en la clínica disminuyó drásticamente poco después de que comenzara la inscripción; por lo tanto, finalizamos la inscripción después de que se completó un grupo focal por sugerencia del personal clínico asociado. Ningún participante optó por usar una cuenta anónima de WhatsApp después de recibir el mensaje directo que describe los riesgos potenciales de usar su número personal de WhatsApp. Era posible inscribirse en el estudio incluso si un participante potencial no tenía una dirección de correo electrónico: para un participante, el coordinador del estudio le indicó que se inscribiera, luego vinculó manualmente al participante al cuestionario al final del grupo focal y le envió su tarjeta como captura de pantalla a través de WhatsApp, en lugar de por correo electrónico.

Las mujeres participaron consistentemente en el grupo focal durante tres días (es decir, todos los participantes compartieron al menos dos respuestas distintas por día [rango: 2-8], ya sea en respuesta a una pregunta de la moderadora o un comentario de un compañero participante). La frecuencia, la exhaustividad y el momento de las respuestas a las preguntas parecen ser más altos en las primeras dos horas después de que el moderador del grupo comenzó la sesión diaria. Cada persona proporcionó al menos una respuesta por día; el retraso más largo entre el inicio diario y la respuesta más lenta del participante fue de aproximadamente dos horas y media. Las mujeres que comenzaron a responder más tarde en el día a menudo comenzaron respondiendo directamente a las preguntas iniciales del moderador y no se sintieron obligados a saltar a las preguntas que otros miembros del grupo estaban discutiendo. Las preguntas en los mensajes que se abrieron pero que no dieron como resultado respuestas fueron repetidas por la moderadora del grupo más adelante en la conversación. El tono general de la conversación fue informal, como lo demuestra el uso continuo de jerga y abreviaturas de Internet y texto utilizadas comúnmente por hispanohablantes de origen mexicano. Dos participantes elaboraron respuestas usando emojis como estos 😱😱😳😳🤔🤔 para expresar miedo (es decir, preocuparse por la exposición al Zika a través de las picaduras de mosquitos) o estos 🙈🙈 para comunicar la incomodidad que explica por qué a los hombres no les gusta usar condones.

La duración media de las respuestas a las preguntas del grupo focal fue de 14 palabras (rango: 4-66, rango intercuartil: 9, 22). Las respuestas fueron concisas y demostraron una comprensión clara del aviso; ninguna respuesta se desvió del tema de un mensaje dado, incluidas las respuestas más largas. Se observó evidencia de que las participantes expresaban respuestas complejas y reflexivas cuando las mismas ideas o cláusulas relacionadas con el mismo mensaje fueron compartidas en mensajes consecutivos (hasta tres seguidos) por el mismo encuestado.

*Corroborando los datos del cuestionario:*

Los datos del cuestionario de REDCap compartidos por las participantes individuales ampliaron la informacion para la interpretación de las respuestas del grupo focal porque los datos estaban vinculados a las participantes por correo electrónico. Cuatro de los cinco participantes respondieron al cuestionario opcional, lo que indica que la carga adicional de completar el cuestionario basado en la web probablemente no fue abrumadora para la población. Debido a que el tamaño de la muestra era tan pequeño, resumimos las tendencias demográficas y de respuesta más relevantes en lugar de informar qué respuestas de el cuestionario se asociaron con participantes individuales: de los cuatro encuestados, tres estaban actualmente embarazadas y tres tenían al menos un nacimiento vivo anterior; las cuatro estaban casadas ​​y vivían con su cónyuge; dos participantes indicaron que buscan información "todo el tiempo" en el Internet que incluye información de salud, como búsquedas de síntomas, mientras que un tercero informó que completó dichas búsquedas varias veces a la semana; tres participantes dijeron que usan las redes sociales varias veces al día o más, aunque nadie dijo que conocen o interactúan regularmente con extraños en línea; dos encuestados indicaron que tienen menos poder en la relación sexual que sus parejas masculinas primarias, y los otros dos indican un poder igual con sus parejas. La mediana de edad fue de 29 años; todos las participantes habían terminado al menos la escuela secundaria (escuela secundaria); solo uno estaba trabajando actualmente.

*Tema 1: conocimiento del zika:*

Los participantes del grup focal estuvieron de acuerdo en que el Zika puede influir en los bebés nacidos de mujeres infectadas ("es un virus transmitido por mosquitos y, desafortunadamente, afecta principalmente al bebé cuya sangre se infecta con el virus"), pero tenían un conocimiento mixto sobre cuáles podrían ser los efectos ( por ejemplo, el zika "causa parálisis en los bebés"). Los participantes sabían sobre la transmisión del Zika transmitida por mosquitos, pero ninguno había oído hablar de la transmisión sexual del virus. Hubo una percepción general de que todos en su comunidad del sur de Arizona están preocupados dado que la exposición a la picadura de mosquito es muy común a nivel local.

*Respondent C: A mi en lo personal si me preocupa mucho. Creo q a todos. Por eso trato de siempre usar manga larga y lantalom [sic] aparte q siempre llevo repelente de mosquitos en mi bolso.*

*Respondent C: *pantalon*

*Respondent A: Yo tampoco miro moskitos dentro d mi hogar y como casi no salgo evitó mucho xk vivo a un lado d un parke y evitamos dejar aguas en botes o estancadas x lo mismo k no se junten mas moscos*

Las participantes informaron que usaban repelente y limpiaban el agua estancada como sus métodos principales para evitar la exposición al virus durante el embarazo.

*Tema 2: Salud sexual y reproductiva:*

Cuando se les preguntó acerca de las recomendaciones médicas hipotéticas para retrasar el embarazo debido al riesgo de Zika, las mujeres dudaron en decir que estarían dispuestas a hacerlo por un período de tiempo indefinido:

*Moderator: En algunos países los doctores recomiendan que no queden embarazadas porque no hay tratamiento para el zika, que harían si su doctor les dijera que eviten quedar embarazadas ?*

*Respondent B: Si en verdad quisiera tener un bebé me cuidaría lo más posible de los mosquitos... y lo pensaría mucho para ver los pros y cons*

*Moderator: Qué tal si dice que se esperen 6 meses?*

*Respondent B: Entonces si me espero 🙂*

*Respondent E: Si el dr lo recomienda creo que debemos hacer caso ya que ellos son los que saben*

Los intergrantes del GF dijeron que no habían hablado con sus médicos sobre el Zika y que sus conversaciones sobre el riesgo del Zika con sus parejas sexuales primarias eran exclusivamente sobre las picaduras de mosquitos, no sobre la transmisión sexual. Cuando se le preguntó sobre el posible inicio del uso del condón durante el embarazo, la confianza percibida fue consistentemente alta entre todas ellas; dos sugirieron que si su pareja era reacia a usar un condón, le mostrarían fotos de bebés nacidos con microcefalia después de la exposición materna al Zika para cambiar de opinión. Los participantes dijeron que no conocían a ninguna mujer cuyas parejas se enojarían o sospecharían si se les pidiera usar un condón durante el embarazo, y además informaron que no habría barreras para obtener condones:

*Respondent A: Creo k nada ps ala pareja ps no le gusta pero a yo pienso k si se trata d cuidarse y d salud eso sale sobrando primero la salud d ambos si esta embarazada ps mas d la d el bebé y si uno esta tratando d salir embarazada ps con musho mas consiencia y cuidarnos para si*

*llegara asalir ps todo marche bien primero dios*

*Tema 3: Uso de tecnología y preferencias:*

Al discutir las preferencias para recibir información sobre el Zika de un profesional de la salud, la mayoría de las participantes indicaron que preferirían que un médico explicará los métodos de prevención en persona. Sin embargo, hubo respuestas encontradas sobre si algunas mujeres que conocían preferirían obtener su información de las noticias, de sus amigos o de Internet. Los participantes comentaron sobre una preocupación compartida de recibir información falsa por Internet e indicaron que preferían hablar con un profesional de la salud, aunque no necesariamente en persona:

*Respondent A: Yo si miro o escucho d algo k se está escuchando musho o algo asi en mi siguiente sita selo comento ami doctora*

**Discusión**

El grupo focal piloto de WhatsApp involucró de manera consistente a todos los participantes durante varios días, obtuvo respuestas sobre temas delicados, incluyó interacciones de los participantes que asemajaban a las que se ven en los grupos tradicionales e involucró en privado a una muestra de mujeres latinas de habla hispana que generalmente dudan de participar en una investigación. El uso de una encuesta en línea suelta para recopilar datos sobre el perfil demográfico, conocimiento y actitudes de frente al Sika amplió la la información sin aumentar la carga para los participantes. Aunque el grupo de enfoque proporcionó una visión inicial de las mejores prácticas metodológicas (**Tabla 1**), la identificación positiva de los participantes (es decir, la confirmación de que los afiliados son quienes dicen ser) sigue siendo un desafío que puede ser motivo de mayor preocupación en un estudio similar donde el área temática es más sensible o la población es más vulnerable a eventos adversos si se viola su confidencialidad. WhatsApp muestra una promesa inicial como plataforma de grupo focal en una población que ya usa la aplicación regularmente.

*Fortalezas y debilidades:*

Además de la premisa de que un enfoque basado en WhatsApp para la entrega de grupos focales podría involucrar a una población reacia, identificamos varias ventajas en el transcurso de este estudio piloto (**Tabla 2**). Por ejemplo, el costo total del grupo piloto se limitó a los costos de impresión, el tiempo de la investigadora y el costo por participante de las tarjetas de regalo (hasta $15 por persona en este estudio); no hubo costos por el alquiler una habitación, el alquiler del equipo, los refrigerios, el transporte, la transcripción, la compra de software u otros componentes que se incurren con frecuencia para las discusiones grupales en persona. Sin embargo, las debilidades que identificamos pueden merecer una consideración adicional para la implementación futura a mayor escala o con poblaciones más vulnerables. Por ejemplo, cuando un solo individuo no respondió a una pregunta, no fue posible distinguir entre la falta de respuesta de no tener una opinión o no entender la pregunta. Este problema podría mitigarse marcando directamente a un participante que no responde en un mensaje de seguimiento para verificar su comprensión.

Mientras este piloto duró tres días para coincidir con los tres temas principales de investigación, se justifican pruebas adicionales para identificar la duración óptima de un grupo focal de WhatsApp y es probable que varíe entre poblaciones y temas. Los temas se superpusieron orgánicamente en otros días más allá de su enfoque previsto, aunque esto también se esperaría en un grupo focal tradicional en persona. Las participantes demasiado vocales ocurren en todos los tipos de investigación de grupos focales, por lo que en un entorno de chat puede ser que la primera voz que se escuche sea también la más dominante, o que simplemente se perciba como tal debido al formato. Como se señaló, hubo una demora de hasta varios minutos entre el momento en que varios participantes abrieron y leyeron una pregunta y el momento en que se recibió la primera respuesta, lo que indica que los primeros en responder también fueron los más entusiastas y se corresponden con las voces dominantes que surgen en grupos focales tradicionales. La etiqueta de conversación basada en chat varía entre plataformas, así como entre poblaciones, por lo que la observación continua de este fenómeno está justificada para estudios futuros utilizando WhatsApp como se describe en la presente investigación.

Aunque no identificamos ningún problema de seguridad relacionado con violaciones de la privacidad en nuestro piloto, algunos problemas potenciales merecen consideración si WhatsApp se usa para grupos focales en otras poblaciones. A los participantes se les asignó la tarea de determinar su nivel de privacidad en el estudio (por ejemplo, la decisión de usar una foto de perfil de su rostro, permitiendo que su número de teléfono sea visto por otros miembros del grupo, usando la aplicación para discutir temas delicados en un dispositivo móvil que un no participante podría acceder potencialmente), así como garantizar la privacidad de otros participantes. La divulgación de información personal en este estudio fue la inclusión de preguntas sobre las experiencias actuales de violencia de pareja; sin embargo, después de que los participantes enviaron esta encuesta, no fue posible para ellas (o sus parejas íntimas) volver a acceder a las respuestas que enviaron. Los temas delicados discutidos en este piloto probablemente no crearon un riesgo significativo de violaciones de privacidad o confidencialidad anticipadas o no anticipadas, este puede no ser el caso en subpoblaciones más pequeñas donde los participantes tienen más probabilidades de conocerse entre sí o donde el intercambio inapropiado de información crea un peligro de indiscreción para otros participantes.

*Implicaciones para uso futuro:*

Investigaciones han identificado cada vez más los usos intencionales y no intencionales de WhatsApp para la comunicación de la salud.^30^ La mayoría de las evaluaciones formales de los grupos de WhatsApp en la literatura muestran su uso entre los proveedores de atención médica,^31^ un ensayo encontró que los debates de texto grupales moderados en WhatsApp reducción de la recaída por fumar en comparación con los panfletos.^32^ Sin embargo, WhatsApp también ha sido un medio para la difusión de información errónea grave, incluyendo la transmisión del Zika (por ejemplo, rumores sobre conspiraciones gubernamentales^33,34^) y la seguridad de las vacunas,^35^ lo que indica una brecha en la cobertura de educación de salud pública que podría aprovecharse positivamente creando información específica de WhatsApp a partir de fuentes demostrablemente autorizadas.

Hasta donde sabemos, nuestro estudio piloto es el primer uso de WhatsApp para discusiones de grupos focales. El potencial para ampliar el acceso a las subpoblaciones es un paso importante en la recopilación de datos. Para algunas poblaciones globales, el uso de datos puede ser un gasto prohibitivo, especialmente cuando el Wi-Fi no es común; sin embargo, WhatsApp usa muy pocos "datos" en comparación con otras aplicaciones, lo que en gran parte explica su éxito a nivel mundial. Sin embargo, el uso de datos sigue siendo una consideración importante para el diseño del estudio, que a menudo se puede mitigar mediante el reembolso de los participantes con crédito telefónico o equivalentes de efectivo.

*Direcciones futuras:*

Más allá de los grupos focales o las entrevistas individuales en profundidad, WhatsApp podría usarse para entregar información educativa estructurada a los grupos objetivo. Por ejemplo, la población involucrada en este estudio piloto puede beneficiarse de los mensajes estructurados sobre el comportamiento del Zika, las mejores prácticas para evitar las picaduras de mosquitos o las formas de alentar a una pareja a usar un condón durante el embarazo. Sin embargo, los participantes en nuestro grupo focal de WhatsApp prefirieron recibir información sobre el zika directamente de un proveedor de atención médica, lo que puede indicar la necesidad de reclutar trabajadores de salud comunitarios capacitados (por ejemplo, promotoras) que puedan demostrar su autoridad sanitaria a los participantes antes de la intervención.

*Conclusiones:*

Este estudio piloto proporciona una plantilla para usar WhatsApp para la organización del grupo focal, así como evidencia inicial de que WhatsApp es un medio factible y de bajo costo para una recopilación eficiente de información cualitativa eficiente. Los métodos innovadores para la recopilación de datos a distancia tienen una gran demanda durante las restricciones relacionadas con COVID-19 sobre los métodos en persona y la baja disponibilidad de fondos para investigación también puede presagiar un mayor uso en el futuro. Se necesitan pruebas adicionales con una gama más amplia de poblaciones y temas para ampliar la comprensión de los riesgos y beneficios tanto para los investigadores como para los participantes. Más allá de los grupos focales, WhatsApp tiene un gran potencial para su uso en la investigación y la implementación de la promoción de la salud entre las poblaciones globales con acceso a teléfonos inteligentes, especialmente donde los profesionales de la salud participan.

Conflictos de intereses: los autores no tienen conflictos que declarar.


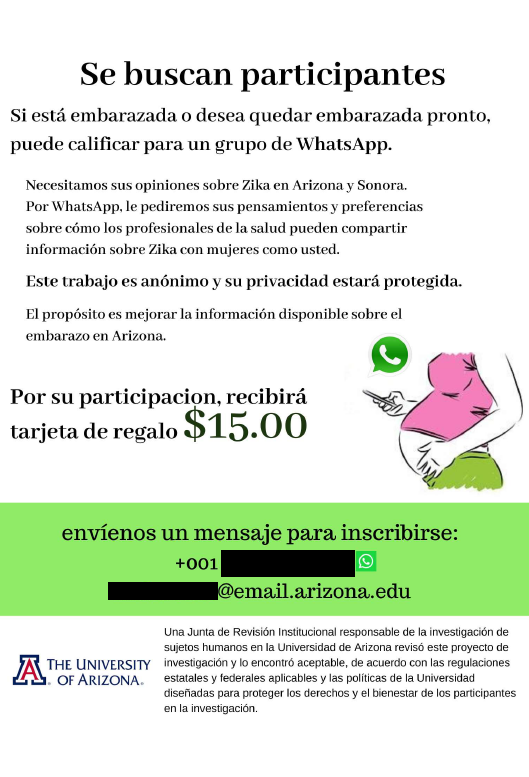


Figura 1: Folleto de reclutamiento para una prueba piloto de WhatsApp como plataforma de grupo focal.


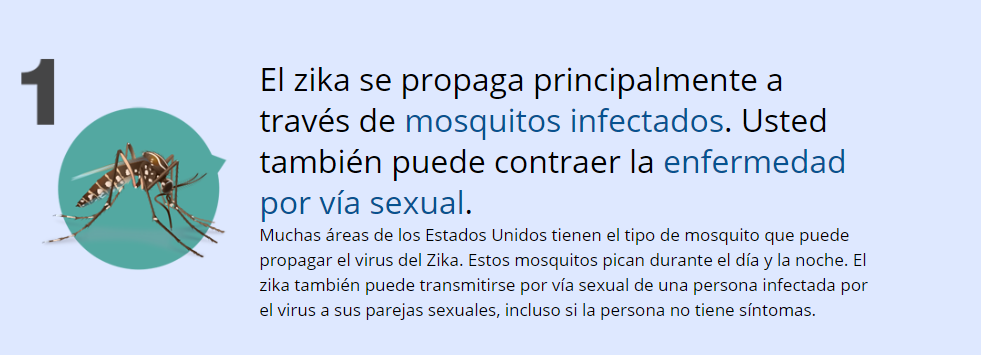


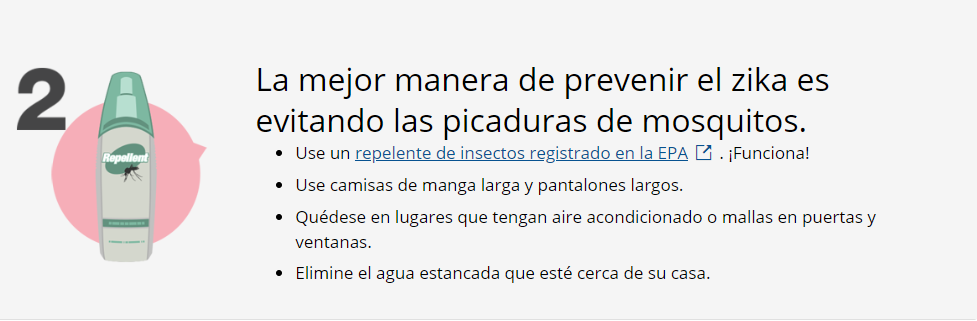


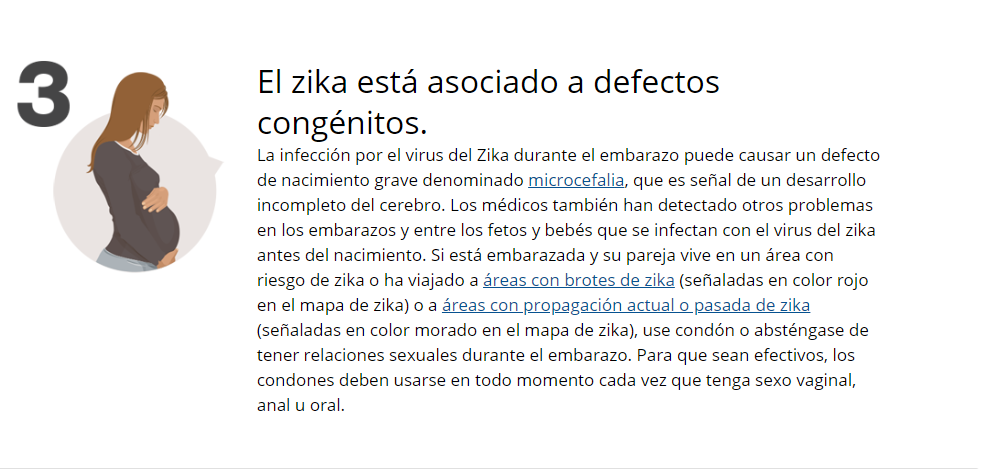


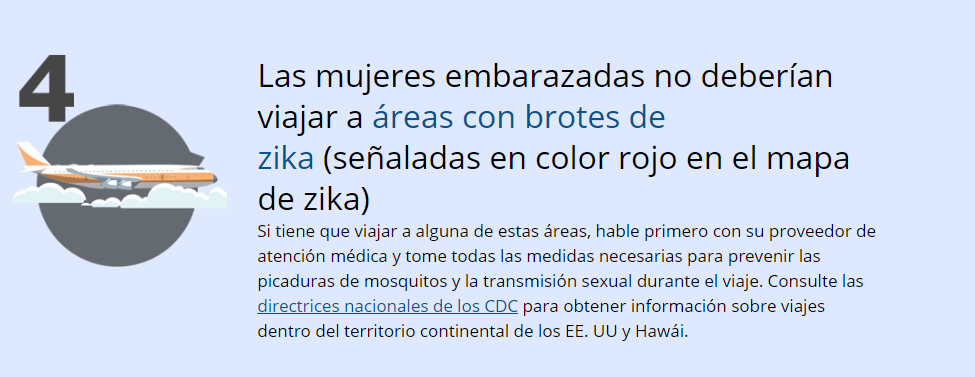

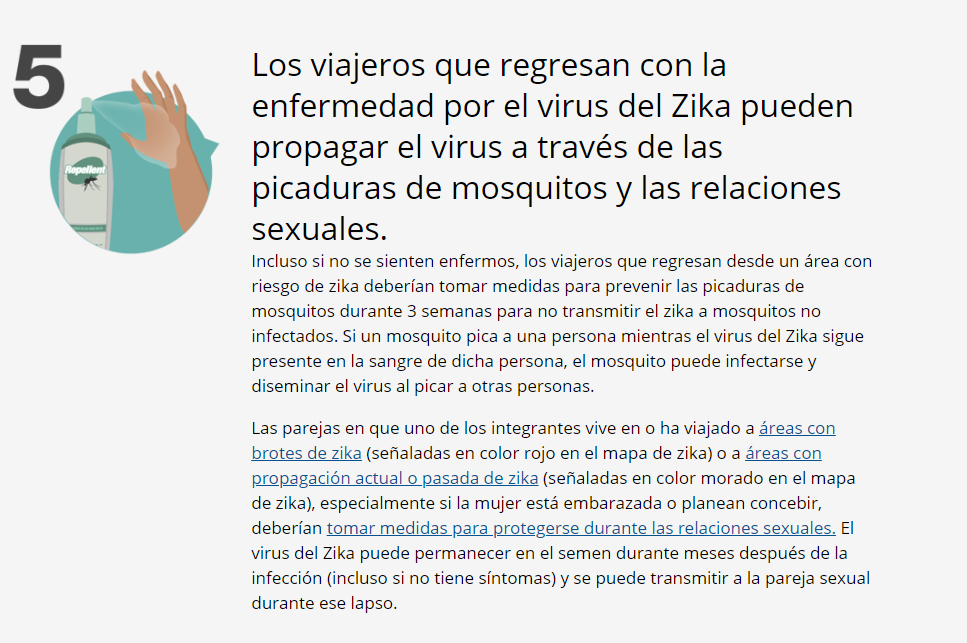


Figura 2: Mensajes informativos compartidos como imágenes con los participantes en un grupo focal piloto de WhatsApp (fuente: CDC)

Tabla 1. Conclusiones de las mejores prácticas de una prueba piloto de WhatsApp como plataforma de grupo focal:

| - Un grupo pequeño (5-7 participantes) trabajó bien en la plataforma, aunque otra literatura soporta grupos en línea más grandes (10-12 participantes); se necesitan más estudios. |
| --- |
| - La realización del grupo focal durante varios días pareció evitar la fatiga de la participación entre las participantes , que respondieron mejor a las preguntas al principio del día en comparación con las preguntas formuladas más tarde en el día. |
| - Los investigadores del estudio no deben vincular la información del estudio a sus números de teléfono personales / cuentas de WhatsApp como sea posible, ya que los participantes usan sus números de teléfono con otros medios en línea (por ejemplo, Facebook), lo que podría comprometer su privacidad. |
| - La carga del uso de “data” en la población probablemente variará, pero debe tenerse en cuenta en el diseño del estudio. |

Tabla 2: Pros y contras de WhatsApp como plataforma de grupo focal identificada en un estudio piloto:

| Pros | Contras |
| --- | --- |
| - Costos de implementación bajos o nulos - Los participantes no necesitan viajar, hacer planes de cuidado de niños o ajustar sus horarios diarios para responder activamente a la discusión del grupo focal. - Los participantes ya usan WhatsApp y no necesitan instalar un nuevo programa o adoptar un nuevo comportamiento para ser participantes activos y exitosos. | - Los participantes no están familiarizados con el enfoque de investigación y es posible que se necesite capacitación adicional para reclutadores para garantizar una explicación precisa del estudio antes de que los participantes se inscriban - La garantía de privacidad está limitada en la medida del cumplimiento de cada participante con las pautas de privacidad, aunque si este riesgo es mayor que los riesgos similares planteados por la investigación en persona depende del área temática del estudio - Las personas altamente vulnerables tienen menos probabilidades de tener teléfonos inteligentes en muchas poblaciones globales y pueden ser excluidas inadvertidamente de las muestras |

Apéndice 1: instrucciones para los participantes para configurar un número de teléfono anónimo en WhatsApp

Para sistemas Android e iOS:

1. Asegúrese de que WhatsApp ya esté instalado.
2. Cree y verifique un número de teléfono alternativo con Google Voice (https://voice.google.com/) siguiendo las instrucciones proporcionadas por Google (disponible en varios idiomas). Debe tener o crear una cuenta con Google para configurar esto. Asegúrese de que los mensajes de texto enviados a su número de Google Voice se reenvíen a su teléfono.

Para Android:

1. Abra la subcarpeta de funciones avanzadas en la configuración. Seleccione la opción para Dual Messenger. Active la opción de mensajería dual para que WhatsApp cree una segunda versión de la aplicación. Siga las instrucciones en pantalla.
2. Abra la versión secundaria recién instalada de WhatsApp. Cuando se le solicite iniciar sesión, hágalo con el número de teléfono de Google Voice. Use la información del texto de confirmación para iniciar sesión.
3. Alternar entre ambas versiones de WhatsApp sin cerrar sesión en la cuenta principal.

Para iOS:

1. Salga de WhatsApp.
2. Inicie una sesión en WhatsApp con el número de teléfono de Google Voice. Use la información del texto de confirmación para iniciar sesión.
3. No es posible alternar entre dos cuentas de WhatsApp en un iPhone. Cerrar sesión y entre cuentas es el único método conocido para usar un número anónimo.

Se espera que en muchas poblaciones mundiales posea y administre múltiples tarjetas SIM, por lo que proporcionar a los participantes una nueva tarjeta SIM puede ser una solución efectiva para garantizar la privacidad del número de teléfono.

**References**

1. Fox FE, Morris M, Rumsey N. Doing synchronous online focus groups with young people: Methodological reflections. *Qualitative health research.* 2007;17(4):539-547.

2. Reid DJ, Reid FJ. Online focus groups: An in-depth comparison of computer-mediated and conventional focus group discussions. *International journal of market research.* 2005;47(2):131-162.

3. Welch V, Petkovic J, Pardo JP, Rader T, Tugwell P. Interactive social media interventions to promote health equity: an overview of reviews. *Health promotion and chronic disease prevention in Canada: research, policy and practice.* 2016;36(4):63.

4. Stewart DW, Shamdasani P. Online focus groups. *Journal of Advertising.* 2017;46(1):48-60.

5. Schneider SJ, Kerwin J, Frechtling J, Vivari BA. Characteristics of the discussion in online and face-to-face focus groups. *Social science computer review.* 2002;20(1):31-42.

6. Han J, Torok M, Gale N, et al. Use of web conferencing technology for conducting online focus groups among young people with lived experience of suicidal thoughts: mixed methods research. *JMIR mental health.* 2019;6(10):e14191.

7. Tates K, Zwaanswijk M, Otten R, et al. Online focus groups as a tool to collect data in hard-to-include populations: examples from paediatric oncology. *BMC Medical Research Methodology.* 2009;9(1):15.

8. Skierkowski D, Wood RM. To text or not to text? The importance of text messaging among college-aged youth. *Computers in Human Behavior.* 2012;28(2):744-756.

9. Lim MS, Sacks-Davis R, Aitken CK, Hocking JS, Hellard ME. Randomised controlled trial of paper, online and SMS diaries for collecting sexual behaviour information from young people. *Journal of Epidemiology & Community Health.* 2010;64(10):885-889.

10. Schober MF, Conrad FG, Antoun C, et al. Precision and disclosure in text and voice interviews on smartphones. *PloS one.* 2015;10(6).

11. Berard B. I second that emoji: The standards, structures, and social production of emoji. *First Monday.* 2018.

12. Neviarouskaya A, Prendinger H, Ishizuka M. Analysis of affect expressed through the evolving language of online communication. Paper presented at: Proceedings of the 12th international conference on Intelligent user interfaces2007.

13. Biedermann N. The use of Facebook for virtual asynchronous focus groups in qualitative research. *Contemporary nurse.* 2018;54(1):26-34.

14. Leavy P. *The Oxford handbook of qualitative research.* Oxford University Press, USA; 2014.

15. Barratt MJ. The efficacy of interviewing young drug users through online chat. *Drug and Alcohol Review.* 2012;31(4):566-572.

16. Diaz VA. Research involving Latino populations. *The Annals of Family Medicine.* 2005;3(5):470-471.

17. Statista. Number of smartphone users in Mexico from 2015 to 2022 (in millions). 2017; <https://www.statista.com/statistics/270970/number-of-smartphone-users-mexico/>.

18. Montag C, Błaszkiewicz K, Sariyska R, et al. Smartphone usage in the 21st century: who is active on WhatsApp? *BMC research notes.* 2015;8(1):331.

19. Nouwens M, Griggio CF, Mackay WE. " WhatsApp is for family; Messenger is for friends" Communication Places in App Ecosystems. Paper presented at: Proceedings of the 2017 CHI conference on human factors in computing systems2017.

20. Mudliar P, Rangaswamy N. Offline strangers, online friends: Bridging classroom gender segregation with whatsapp. Paper presented at: Proceedings of the 33rd Annual ACM Conference on Human Factors in Computing Systems2015.

21. Lin K-Y, Lu H-P. Why people use social networking sites: An empirical study integrating network externalities and motivation theory. *Computers in human behavior.* 2011;27(3):1152-1161.

22. Coyle CL, Vaughn H. Social networking: Communication revolution or evolution? *Bell Labs technical journal.* 2008;13(2):13-17.

23. Casal M. Internal report Border Infectious Disease Surveillance Program – Dec. 31st, 2018, Zika Virus Location Report. In: Services ADoH, ed2018.

24. Finer LB, Kost K. Unintended pregnancy rates at the state level. *Perspectives on Sexual and Reproductive Health.* 2011;43(2):78-87.

25. Davila YR. Influence of abuse on condom negotiation among Mexican-American women involved in abusive relationships. *Journal of the Association of Nurses in AIDS Care.* 2002;13(6):46-56.

26. Harris PA, Taylor R, Thielke R, Payne J, Gonzalez N, Conde JG. Research electronic data capture (REDCap)—a metadata-driven methodology and workflow process for providing translational research informatics support. *Journal of biomedical informatics.* 2009;42(2):377-381.

27. Corden A, Sainsbury R. *Using verbatim quotations in reporting qualitative social research: researchers' views.* University of York York; 2006.

28. Matsuda Y, McGrath JM, Jallo N. Use of the sexual relationship power scale in research: An integrative review. *Hispanic Health Care International.* 2012;10(4):175-189.

29. Costa JJ, Matos AP, Rosario MdP, Salvador C, Luz Vale-Dias Md, Zenha-Rela M. Evaluating use and attitudes towards social media and ICT for portuguese youth: the MTUAS-PY scale. 2016.

30. Kamel Boulos MN, Giustini DM, Wheeler S. Instagram and WhatsApp in health and healthcare: An overview. *Future Internet.* 2016;8(3):37.

31. Mars M, Scott RE. WhatsApp in Clinical Practice: A Literature. *The Promise of New Technologies in an Age of New Health Challenges: Selected Papers from Global Telehealth 2016.* 2016;231:82.

32. Cheung YTD, Chan CHH, Lai C-KJ, et al. Using WhatsApp and Facebook online social groups for smoking relapse prevention for recent quitters: a pilot pragmatic cluster randomized controlled trial. *Journal of medical Internet research.* 2015;17(10):e238.

33. Kou Y, Gui X, Chen Y, Pine K. Conspiracy talk on social media: collective sensemaking during a public health crisis. *Proceedings of the ACM on Human-Computer Interaction.* 2017;1(CSCW):61.

34. Vijaykumar S, Nowak G, Himelboim I, Jin Y. Managing social media rumors and misinformation during outbreaks. *American journal of infection control.* 2018;46(7):850.

35. Palanisamy B, Gopichandran V, Kosalram K. Social capital, trust in health information, and acceptance of Measles–Rubella vaccination campaign in Tamil Nadu: A case–control study. *Journal of postgraduate medicine.* 2018;64(4):212.

**Abreviaturas**

REDCap Research Electronic Data Capture / Captura electrónica de datos

SMS Servicio de mensajes cortos

EE.UU. Los Estados Unidos
